# Supplementary material for: Striatal dopamine D2/D3 receptor regulation of human reward processing and behaviour
Source: Nat Commun. 2025 Feb 21;16:1852. doi: 10.1038/s41467-025-56663-7 (PMC11845780; doi:10.1038/s41467-025-56663-7)
Supplement: Supplementary file 1 — Supplementary Information [file 41467_2025_56663_MOESM1_ESM.pdf]

Striatal dopamine D2/D3 receptor regulation of human reward processing and behaviour: Supplementary information

Supplementary Methods

Screening and safety procedures

At the screening visit, subjects underwent a full physical examination including vital signs, psychiatric, medical and medication history, electrocardiogram (ECG), blood sampling (screening bloods: full blood count, liver function, urea and electrolytes, C-reactive protein), urine drug of abuse testing, and urine pregnancy testing (females). All of these procedures were repeated at all five study appointments, although blood samples were analysed for drug levels instead of the tests detailed above after the screening appointment. For the entire duration of the study, participants were advised to abstain from alcohol and recreational drugs, and females were advised to use effective contraception, with the latter two verified by urine testing as detailed above.

Sequence generation and allocation concealment

Upon enrolment, subjects were randomised to treatment order (amisulpride or placebo first in arm 1, aripiprazole or placebo first in arm 2). Allocation of drug order was determined by the Latin Square design such that the planned sequence of treatments was balanced for order. The study clinicians and subjects were blind to the treatment order, as an independent researcher allocated subject IDs.

Amisulpride/aripiprazole were encapsulated and lactose powder was used to produce placebo capsules which were identical in shape, size, colour and number to the capsules containing active drug. Placebo and active capsules were dispensed in identical pill bottles.

MRI acquisition

Structural and functional MRI images were acquired on a 3T Siemens Magnetom Prisma with a 64-channel head coil. High resolution T1 weighted volumes were acquired using a MP2RAGE sequence (176 slices, FOV read = 256 mm, TR = 5000.0 ms, TE = 2.98 ms, TI 1 = 707 ms, TI 2 =

2500 ms, flip angle 1 = 4°, flip angle 2 = 5°, 1 mm isotropic voxels, band- width = 240 Hz/pixel, echo spacing = 7.1 ms).

For the MID task, at each scanning session, 304 functional volumes were acquired using a BOLD echo planar imaging sequence (FOV read 220mm, 42 slices, slice thickness 3 mm, voxel size 3.4 x 3.4 x 3mm, TR 2400 ms, TE 30 ms). The first six volumes of each functional run were discarded to allow for T1 saturation effects.

Measurement of regional CBF was carried out using a 3D pseudo-continuous ASL (3D-PCASL) sequence and the following parameters: bolus duration= 1800 ms, inversion time= 3600ms, FOV= 220 mm, TR= 4000ms, TE= 13.32ms, slice thickness= 3 mm, resolution 1.7 x 1.7 x 3 mm, acquisition time= 4 minutes 42 s. Eight control-label pairs were used to derive a perfusion-weighted difference image. The sequence included background and fat suppression for optimal reduction of the static tissue signal. The sequence also included a perfusion calibration (M0) scan with TR= 5,000 ms to compute the CBF map in standard physiological units (ml blood/100 g tissue/min).[1, 2]

Monetary incentive delay (MID) task

We used the MID task to assess neural response to reward stimuli. It probes brain activation upon expectation and receipt of monetary reward in a multiple-trial design[3]. The task contains two trial types (24 win trials and 48 neutral trials). Participants are instructed to respond as quickly as possible to a target stimulus, and can win money if they respond quickly enough during win trials. The total amount of money to be won is £7.20, with £0 or £0.30 at stake in each trial. Each trial begins with the presentation of a cue stimulus for 500ms, which denotes whether the trial is a win trial (orange square) or a neutral trial (blue square). Following the cue, there is an anticipation period (interstimulus interval (ISI)) which varies randomly between 2, 3, and 4 seconds. The target stimulus (a white square) is then presented for a variable duration (starting at 300ms, 16.67ms subtracted or added each trial depending on performance in previous trial, range 200-400ms), during which time the subject has to respond. The target hit rate was approximately 50%. Following the target, feedback on the outcome of the trial is presented. The duration of feedback is also dynamic, to ensure that the total duration of the target plus the feedback is 1300ms. Following the feedback, an inter-trial interval (ITI) consisting of a fixation point is presented, which varies randomly between 2.2 and 10.2 seconds in one-second increments, on an approximately Poisson distribution[4].

MID behavioural analysis

Any participant performing more than 2.5 SDs outside the session average in percentage of hits or average reaction time across all response trials was removed from the analysis of that session, in line with previous analyses[5]. Percentage of successful trials, percentage of successful reward trials, overall reaction time, and reaction time to reward trials were compared between the baseline, amisulpride or aripiprazole and placebo conditions using a repeated measures ANOVA in SPSS.

fMRI preprocessing and analysis

Image processing was performed using FSL version 6.00. Anatomical images were pre-processed using the FSL_anat function in FSL to perform bias correction, transformation into a standard stereotactic space (MNI152) and brain extraction using the BET extraction tool. Functional image series were pre-processed with a 100s high-pass filter, head motion correction, 6mm full width at half maximum spatial smoothing and co-registration to the T1-weighted structural image before transformation to standard space.

The times of the stimulus conditions were convolved with a gamma function to simulate the haemodynamic response function. Head-motion parameters were included in the first-level (subject-level) models as nuisance regressors. Temporal derivatives of task-related events and neutral hit and neutral miss explanatory variables were also included as nuisance regressors. We conducted a second-level analysis to calculate the group mean of all baseline scans (regardless of subsequent treatment order or condition) to ensure that the tasks were activating the reward network. We used FSL's FLAME 1 model, thresholded at $Z > 3.1$, using a corrected cluster significance threshold of $p = 0.05$.

Cerebral blood flow (CBF)

Quantified CBF maps for each subject and session were skull-stripped with BET as implemented in FSL, co-registered to their corresponding baseline structural scan, normalized to the study-specific T1-weighted template and subsequently warped to standard MNI space and resampled to a 2 mm3 isotropic resolution using ANTs. Finally, CBF images were spatially smoothed using a 6 mm FWHM Gaussian kernel. Regional CBF was extracted from identical striatal ROIs to the fMRI analysis from the Harvard Oxford subcortical atlas, using the fslmeants function in FSL.

Potential confounding on the BOLD signal from rCBF was assessed using matlab’s fitlme function. The main predictor was the fixed effect of treatment condition (active drug or placebo), and we also included random intercepts and random slopes for each participant, and the fixed effect of caudate rCBF. The code was as follows: fitlme(Data, 'Response~DrugCondition + rCBF + (DrugCondition|subjectID)')

Supplementary Results

Recruitment, adverse effects and sample demographics

Amisulpride: 39 subjects were enrolled in the study, and 25 subjects completed it. Two subjects withdrew consent prior to the baseline visit, therefore 37 subjects received at least one dose of study medications. Four of these subjects withdrew prior to completing the first follow up visit, all due to adverse events – three due to akathisia whilst receiving amisulpride, the other due to anxiety on placebo. Thirty-three subjects therefore completed the baseline assessment and first follow up assessment. Two subjects were lost to follow up prior to the second treatment week, and another due to adverse events during the second treatment week (acute dystonia whilst receiving amisulpride). Thirty subjects therefore completed both treatment conditions, however five subjects were found to have undetectable plasma amisulpride levels at both follow up appointments; these subjects were excluded from all post baseline analyses.

Aripiprazole: Thirty-seven subjects were enrolled in the study, and 25 subjects completed it. Four subjects withdrew consent prior to the baseline visit, and one subject was excluded following the baseline assessments but prior to receiving study medications due to cardiac symptoms reported at the baseline visit but not at screening. Thirty-two subjects therefore received at least one dose of study medications. Four of these subjects withdrew prior to the first follow up visit, three due to adverse events (two: fatigue whilst receiving aripiprazole, one: tonsilitis on placebo) and one was lost to follow up. 28 subjects therefore completed the baseline assessment and first follow up assessment. One subject was lost to follow up prior to the second treatment week, and another due to adverse events during the second treatment week (vomiting whilst receiving aripiprazole). Twenty-six subjects therefore completed both treatment conditions, however one subject was found to have undetectable plasma

aripiprazole levels at both follow up appointments; this subject was excluded from all post baseline analyses. No subjects had detectable plasma aripiprazole/de-hydroaripiprazole levels following the washout period (at the dosing visit).

Table S1: Description of sample. Values are mean (SD) for continuous variables, and frequencies for categorical variables unless otherwise stated.

	Amisulpride (n = 25)	Aripiprazole (n = 25)	Comparison
**Sex**	Female 15 (60%); Male 10 (40%)	Female 14 (56%); Male 11 (44%)	p=0.78
**Age (years)**	26.5 (7.6)	26.7 (8.7)	p=0.93
**Ethnicity**	White 15, Asian 4, Black 1, Mixed/Other 5	White 16, Asian 5, Black 2, Mixed/Other 2	p=0.62
**BMI**	23.6 (3.8)	22.4 (3.5)	p=0.25
**Education**	Postgraduate 6, Undergraduate 13, High School 4, Professional Qualification 1	Postgraduate 9, Undergraduate 6, High School 9, Other 1	p=0.15
**Employment**	Student 19, Employed 6	Student 16, Employed 9	p=0.36
**Treatment order**	Amisulpride first 13 (52%), placebo first 12 (48%)	Aripiprazole first 12 (48%), placebo first 13 (52%)	p=0.78
**Plasma levels (ug/L)**	312.4 (203.0). Range 29-719	Aripiprazole: 95.8 (33.3). Range 35-174	N/A

		Aripiprazole + De-hydroaripiprazole: 126 (31.4). Range 58-193	
**Washout length (days)**	23.6 (13.7). Range 12-64	52.6 (37.6). Range 28-168	N/A

Comparisons between demographic variables were performed using two-sided independent sample t-tests for continuous variables, and chi-squared tests for categorical variables.

Behavioural and motor effects of the D2/D3 receptor antagonist amisulpride

Table S2: Output of mixed effects model (effect of amisulpride on BNSS)

**Predictor**	**Fixed Effect Estimate (β)**	**t**	**p**
Condition: Amisulpride > Placebo	4.62	2.51	0.015*
Order: Placebo first	3.05	1.50	0.14
BNSS score at baseline	0.84	4.59	3.13×10^{-5}*

R^2=0.73

Table S3: Output of mixed effects model (effect of amisulpride on SAS)

**Predictor**	**Fixed Effect Estimate (β)**	**t**	**p**
Condition: Amisulpride > Placebo	1.03	3.12	0.0030*
Order: Placebo first	0.23	1.44	0.16

R^2=0.96, Baseline scores not included in model as all subjects scored 0 at baseline

Table S4: Output of mixed effects model (effect of amisulpride on BARS)

**Predictor**	**Fixed Effect Estimate (β)**	**t**	**p**
Condition: Amisulpride > Placebo	1.48	4.53	$3.63 \times 10^{-5*}$
Order: Placebo first	9.03×10^{-12}	2.41×10^{-6}	1

R^2=1.00, Baseline scores not included in model as all subjects scored 0 at baseline

Table S5: Output of mixed effects model (effect of amisulpride on BNSS expressive subscale)

Predictor	Fixed Effect Estimate (β)	t	p
Condition: Amisulpride > Placebo	2.12	2.89	0.0057*
Order: Placebo first	0.74	0.82	0.41
BNSS score at baseline	0.69	3.08	0.0034*

R^2=0.76

Table S6: Output of mixed effects model (effect of amisulpride on BNSS motivational subscale)

Predictor	Fixed Effect Estimate (β)	t	p
Condition: Amisulpride > Placebo	1.89	1.55	0.13
Order: Placebo first	2.24	1.80	0.077
BNSS score at baseline	0.94	5.44	1.66×10^{-6}*

R^2=0.85

Results reported in tables S2:S6 are two-sided p-values from linear mixed effects models from intention to treat analysis based on all eligible subjects who completed at least one post baseline treatment condition (n=29). The significance of results was unchanged in complete case analysis (n=24):

- BNSS total score: amisulpride vs placebo p value=0.046*
- SAS: amisulpride vs placebo p-value=0.0085*
- BARS: amisulpride vs placebo p-value=0.00025*
- BNSS expressive subscale: amisulpride vs placebo p-value =0.015*
- BNSS motivational subscale: amisulpride vs placebo p-value =0.30

MID task – baseline task activation

Data were available for 64 subjects for the analysis of baseline task effects. One subject was excluded due to technical issues with the MRI acquisition, and five subjects were excluded due to poor task performance (all scored 7% or less).

Win anticipation

At baseline, the contrast of win anticipation > neutral anticipation resulted in widespread activation in the bilateral thalamic nuclei, bilateral striatum (globus pallidus, putamen, caudate), in related cortical reward structures (insula, middle and inferior frontal gyri, frontal pole, anterior and posterior cingulate), and in the cerebellum, temporal, parietal and occipital lobes (Fig S1).

Win outcome

At baseline, the contrast of win hit > win miss resulted in widespread activation in the bilateral thalami, hippocampi, amygdalae, as well as the bilateral striatum (globus pallidus, nucleus accumbens, putamen, caudate), related cortical reward structures (insula, middle and inferior frontal gyri, frontal pole, anterior and posterior cingulate), and the cerebellum, temporal, and occipital lobes (Fig S2).

Fig S1: Baseline task activation in MID task (win anticipation–neutral anticipation). Colour bar indicates z-statistic, n=64.

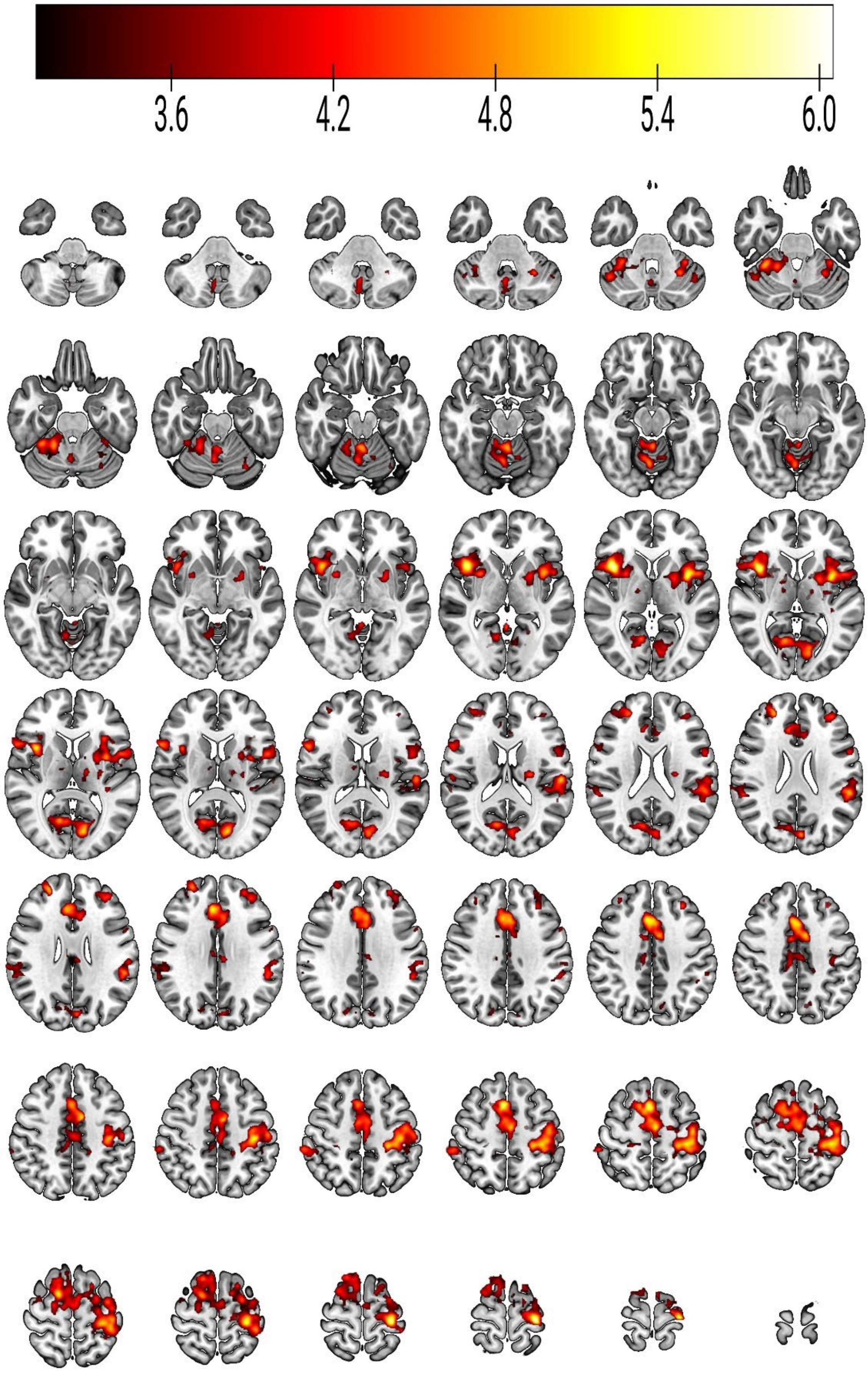

Fig S2: Baseline task activation in MID task (win hit–win miss). Colour bar indicates z-statistic, n=64.

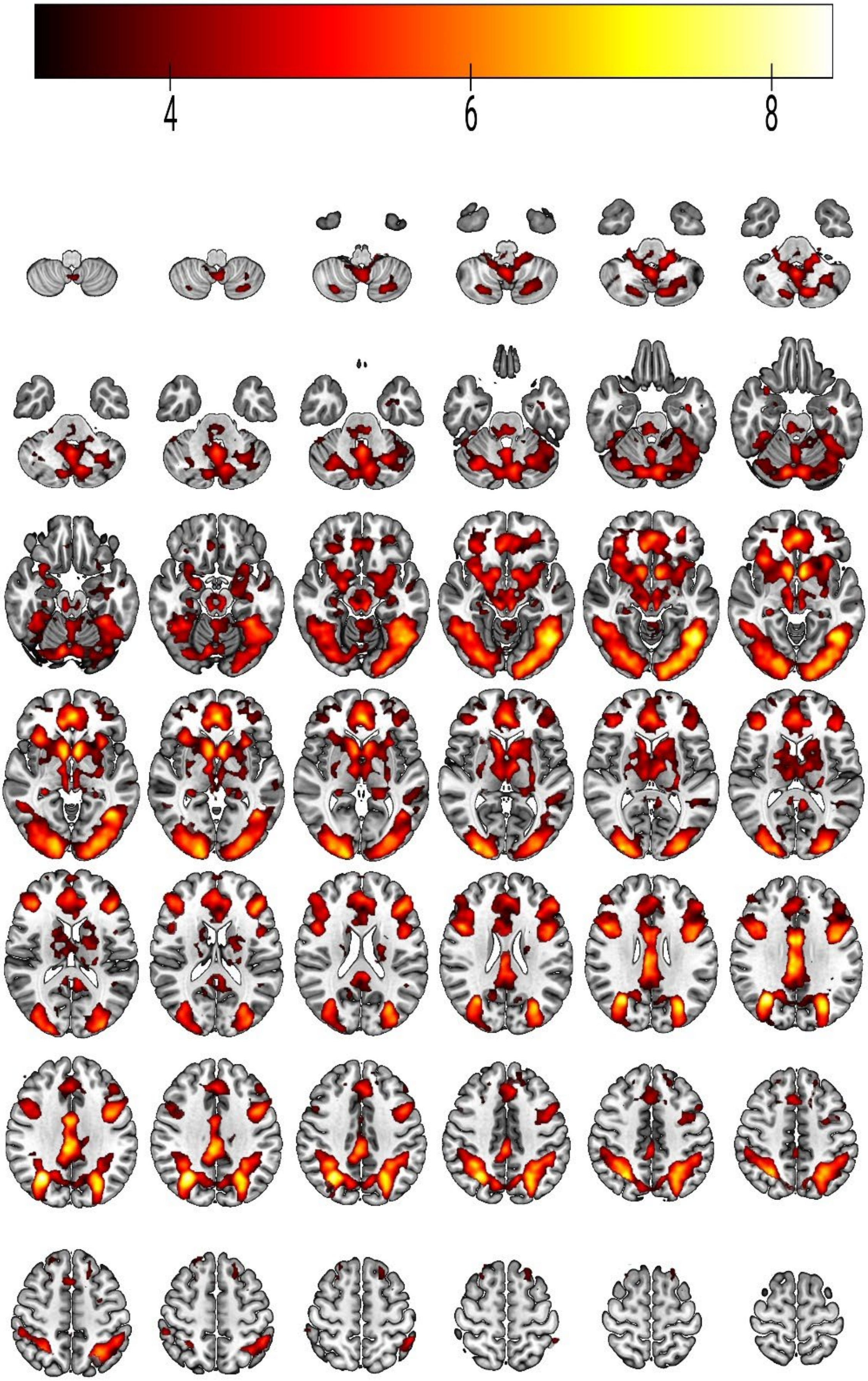

Effects of D2/D3 receptor antagonist amisulpride on reward response

Data were available for 21 subjects for the main comparison of the amisulpride and placebo conditions; one subject terminated the scanning session prior to the completion of the task, two subjects were excluded due to technical issues with the MRI acquisition, and one subject was excluded due to poor task performance, achieving only 12.5% accuracy in the amisulpride condition.

There was no significant effect of either amisulpride or of practice on task performance; there was no difference in overall accuracy, overall reaction time, accuracy in reward trials or reaction time in reward trials between the baseline, amisulpride and placebo conditions:

- Overall accuracy: $F(2, 38)= 0.25$, $p=0.78$
- Reward trial accuracy: $F(2, 38)= 0.63$, $p=0.94$
- Overall reaction time: $F(2, 38)= 0.31$, $p=0.97$
- Reward trial response time: $F(2, 38)= 0.38$, $p=0.63$

Table S7: Striatal effects of amisulpride vs placebo on MID task. n=21

	**Amisulpride mean beta value (SD)**	**Placebo mean beta value (SD)**	**FDR corrected p-value**
**Win anticipation**			
Caudate	-19.87 (58.01)	0.64 (37.02)	p=0.36
Putamen	-5.82 (65.24)	7.4 (30.66)	p=0.36
Nucleus accumbens	-32.65 (86.90)	-3.69 (72.66)	p=0.36
**Win outcome**			
Caudate	-35.9 (200.86)	110.26 (117.81)	p=0.014*
Putamen	39.49 (135.59)	57.29 (109.92)	p=0.56
Nucleus accumbens	-36.17 (258.01)	60.24(151.39)	p=0.23

p-values are from two-sided paired sampled t-tests, with FDR correction for multiple comparisons

Fig S3: Axial slices from whole brain analysis displaying clusters showing significant BOLD reduction on amisulpride compared to placebo in win hit–win miss contrast.
Colour bar indicates z statistic, n=21

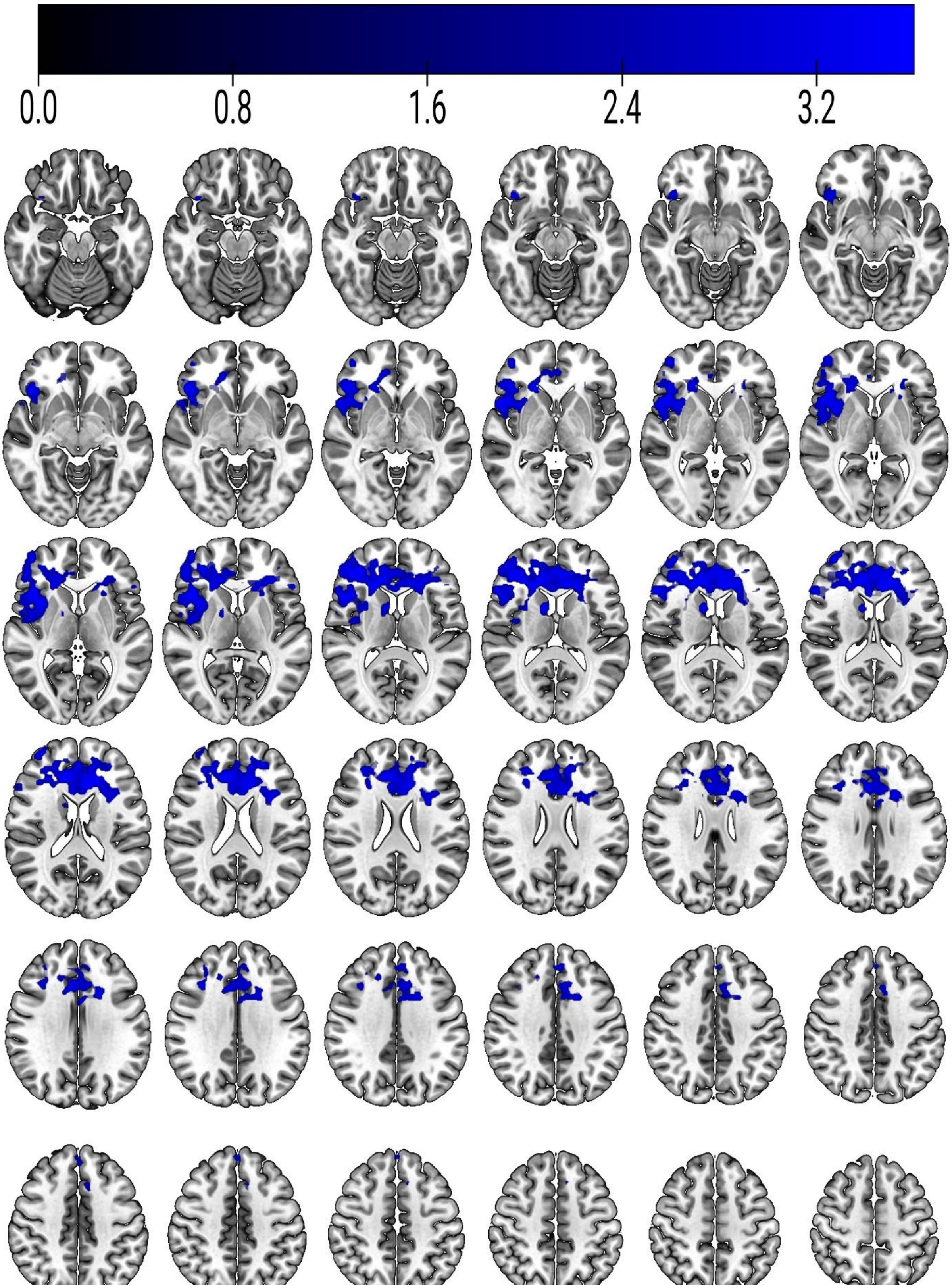

Table S8: Correlations between change in outcome variables from baseline to amisulpride and change in BNSS factors across same time period.

	**Caudate reward outcome**	**Parkinsonian symptoms (SAS)**	**Akathisia (BARS)**
**BNSS total score**	n = 21, rho = -0.492, FDR corrected p-value = 0.041*	n=27, rho=0.617, p=0.0006*	n=27, rho=0.227, p=0.25
1.BNSS expressive factor	n = 21, rho = -0.412, p = 0.064	n=27, rho=0.549, p=0.003*	n=27, rho=0.029, p=0.89
1.1 BNSS alogia factor	n = 21, rho = -0.357, p = 0.11	n=27, rho=0.449, p=0.019*	-
1.2 BNSS blunted affect factor	n=21, rho=-0.202, p=0.38	n=27, rho=0.636, p= 0.0004*	-
2.BNSS motivational factor	n = 21, rho = -0.396, p = 0.076	n=27, rho=0.439, p=0.022*	n=27, rho=0.229, p=0.25
2.1 BNSS avolition factor	n=21, rho=-0.459, p=0.037*	n=27, rho=0.384, p=0.048*	-
2.2 BNSS anhedonia factor	n=21, rho=-0.292, p=0.20	n=27, rho=0.292, p=0.14	-
2.3 BNSS asociality factor	n=21, rho=-0.185, p=0.42	n=27, rho=0.553, p=0.003*	-

p-values are from Spearman’s rho correlations, exploratory analyses uncorrected for multiple comparisons

Behavioural and motor effects of the D2/D3 receptor partial agonist aripiprazole

Table S9: Output of mixed effects model (effect of aripiprazole on BNSS)

Predictor	Fixed Effect Estimate (β)	t	p
Condition: Aripiprazole > Placebo	1.24	1.69	0.10
Order: Placebo first	-0.29	-0.47	0.64
BNSS score at baseline	0.75	3.94	0.00027*

R^2=0.90

Table S10: Output of mixed effects model (effect of aripiprazole on SAS)

Predictor	Fixed Effect Estimate (β)	t	p
Condition: Aripiprazole > Placebo	1.19	3.61	0.00073*
Order: Placebo first	-0.17	-0.63	0.53
SAS score at baseline	1.62	6.55	$3.56x10^{-8*}$

R^2=0.92

Table S11: Output of mixed effects model (effect of aripiprazole on BARS)

Predictor	Fixed Effect Estimate (β)	t	p
Condition: Aripiprazole > Placebo	1.04	3.31	0.0017*
Order: Placebo first	$-5.94x10^{-11}$	$-3.06x10^{-5}$	1.00

R^2=1.00, Baseline scores not included as all subjects scored 0 at baseline

Table S12: Output of mixed effects model (effect of aripiprazole on BNSS expressive subscale)

Predictor	Fixed Effect Estimate (β)	t	p
Condition: Aripiprazole > Placebo	0.65	1.37	0.18
Order: Placebo first	0.24	0.55	0.58
BNSS score at baseline	0.60	4.16	0.00013*

R^2=0.81

Table S13: Output of mixed effects model (effect of aripiprazole on BNSS motivational subscale)

Predictor	Fixed Effect Estimate (β)	t	p
Condition: Aripiprazole > Placebo	0.60	1.51	0.14
Order: Placebo first	0.49	1.98	0.054
BNSS score at baseline	1.41	5.16	4.61×10^{-6}*

R^2=0.92

Results reported in tables S8:S12 are two-sided p-values from linear mixed effects models from intention to treat analysis based on all eligible subjects who completed at least one post baseline treatment condition (n=27). The significance of results was unchanged in complete case analysis (n=25):

- BNSS total score: aripiprazole vs placebo p value=0.12
- SAS: aripiprazole vs placebo p-value=0.0058*
- BARS: aripiprazole vs placebo p-value=0.0017*
- BNSS expressive subscale: aripiprazole vs placebo p-value =0.25
- BNSS motivational subscale: aripiprazole vs placebo p-value =0.12

Effects of D2/D3 receptor partial agonist aripiprazole on reward response

Data were available for 22 subjects for the main comparison of the aripiprazole and placebo conditions; two subjects were excluded due to poor task performance, achieving only 8% accuracy (one in the aripiprazole condition and one on placebo). One subject was excluded due to fast reaction times in the placebo condition, responding more than 2.5 SDs faster than the session average.

There was no significant effect of either aripiprazole or of practice on task performance; there was no difference in overall accuracy, overall reaction time, accuracy in reward trials or reaction time in reward trials between the baseline, aripiprazole and placebo conditions:

- Overall accuracy: $F(2, 40)= 0.42$, $p=0.66$
- Reward trial accuracy: $F(2, 40)= 0.26$, $p=0.77$
- Overall reaction time: $F(2, 40)= 0.58$, $p=0.57$
- Reward trial response time: $F(2, 40)= 0.13$, $p=0.27$

Table S14: Striatal effects of aripiprazole vs placebo during MID task. n=22

	**Aripiprazole mean beta value (SD)**	**Placebo mean beta value (SD)**	**FDR corrected p-value**
**Win anticipation**			
Caudate	3.89 (52.59)	-11.26(50.96)	p=0.42
Putamen	5.69 (51.58)	8.36 (37.69)	p=0.83
Nucleus accumbens	1.92(66.78)	-28.19 (57.21)	p=0.39
**Win outcome**			
Caudate	85.46 (165.06)	58.62 (214.61)	p=0.54
Putamen	84.98 (129.85)	48.26 (172.90)	p=0.54
Nucleus accumbens	98.87 (201.22)	140.09 (145.69)	p=0.54

p-values are from two-sided paired sampled t-tests, with FDR correction for multiple comparisons

Comparison between D2/D3 antagonism and D2/D3 partial agonism

Table S15: Comparison of amisulpride–placebo to aripiprazole–placebo effects on MID task (n = 43)

	**Amisulpride–placebo mean beta value (SD)**	**Aripiprazole–placebo mean beta value (SD)**	**FDR corrected p-value**
**Win anticipation**			
Caudate	-20.52 (68.03)	15.14 (63.90)	p=0.13
Putamen	-13.23 (64.59)	-2.67 (58.74)	p=0.58
Nucleus accumbens	-28.96 (112.90)	30.12 (90.75)	p=0.13
**Win outcome**			
Caudate	-146.19 (211.33)	26.84 (201.99)	p=0.027*
Putamen	-17.80 (138.46)	36.72 (198.54)	p=0.46
Nucleus accumbens	-96.42 (298.06)	-41.22 (266.58)	p=0.53

p-values are from two-sided independent sample t-tests, with FDR correction for multiple comparisons

Fig S4: Axial slices from whole brain analysis displaying clusters showing significantly greater BOLD reduction on amisulpride–placebo compared to aripiprazole–placebo in win hit–win miss contrast. Colour bar indicates z statistic, n=43.

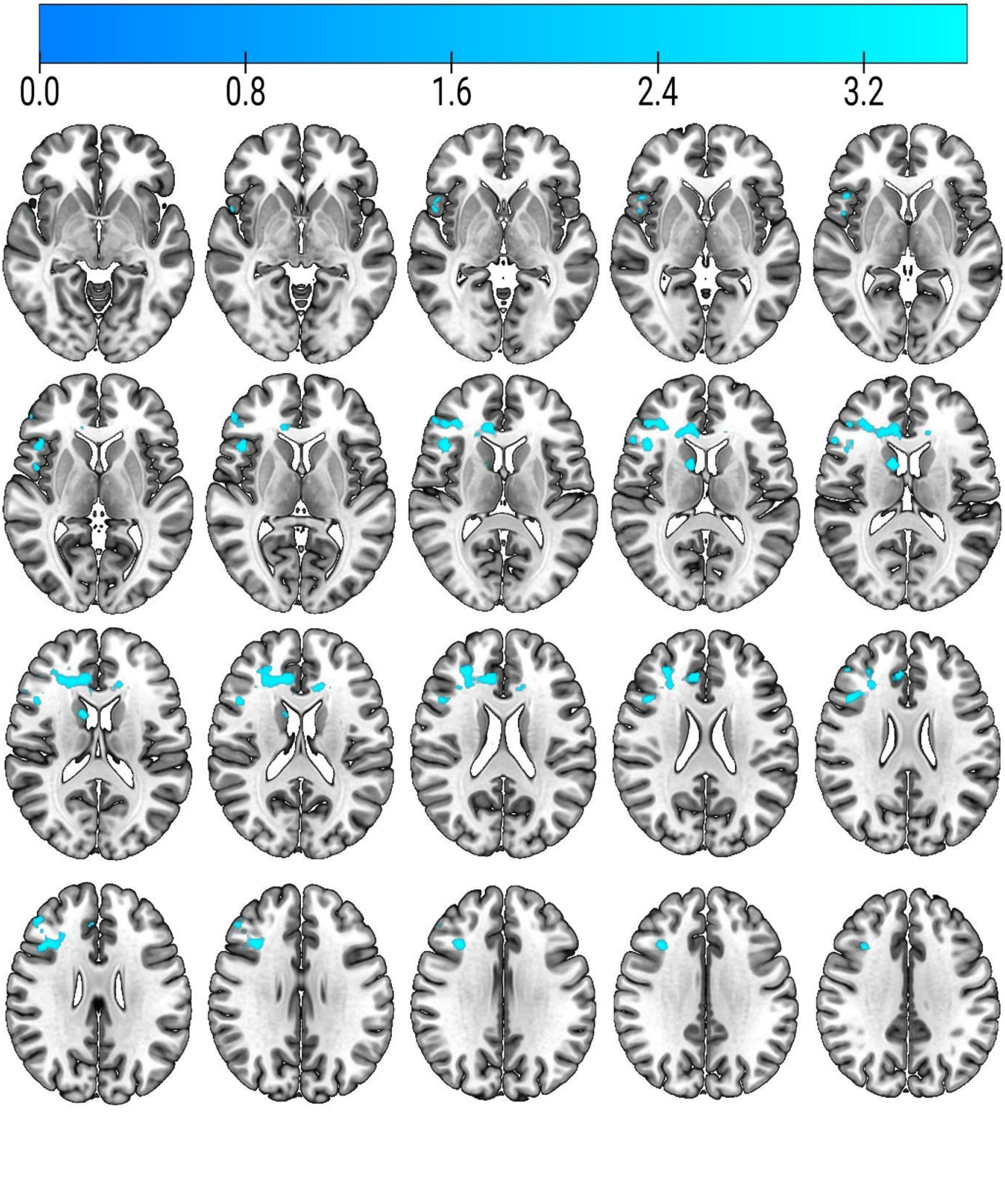

Table S16: Comparison of baseline and placebo values for outcome variables between amisulpride and aripiprazole samples

	Amisulpride mean value (SD)	Aripiprazole sample mean value (SD)	FDR corrected p-value
**Clinical measures**			
Baseline: BNSS Total	1.79 (5.4), n=29	0.67 (1.62), n=27	p=0.31
Baseline: SAS	0.00 (0), n=29	0.15 (0.53), n=27	p=0.25
Baseline: BARS	0.00 (0), n=29	0.00 (0), n=27	N/A
Placebo: BNSS Total	4.15 (7.77), n=26	1.04 (2.01), n=26	p=0.25
Placebo: SAS	0.12 (0.43), n=26	0.46 (1.17), n=26	p=0.25
Placebo: BARS	0.00 (0), n=26	0.00 (0), n=26	N/A
Baseline to Placebo Δ: BNSS	2.15(5.95), n=26	0.35 (1.64), n=26	p=0.25
Baseline to Placebo Δ: SAS	0.12 (0.43), n=26	0.31 (0.84), n=26	p=0.31
Baseline to Placebo Δ: BARS	0.00 (0), n=26	0.00 (0), n=26	N/A
**Win Anticipation**			
Baseline: Caudate (beta values)	8.82 (55.91), n=25	8.68 (76.18), n=25	p=0.99
Baseline: Putamen (beta values)	21.23 (35.82), n=25	25.32 (52.10) , n=25	p=0.98
Baseline: Nucleus accumbens (beta values)	4.55 (68.62), n=25	-3.56 (42.54), n=25	p=0.98
Placebo: Caudate (beta values)	-1.07 (40.73), n=24)	-4.15 (54.35), n=24	p=0.98
Placebo: Putamen (beta values)	6.60 (41.89), n=24	11.98 (38.22), n=24	p=0.98

Placebo: Nucleus accumbens (beta values)	2.22 (74.73), n=24	-17.64 (65.86), n=24	p=0.98
Baseline to Placebo Δ: Caudate (beta values)	4.62 (107.84), n=22	-16.51 (81.33), n=23	p=0.98
Baseline to Placebo Δ: Putamen (beta values)	-6.69 (62.30), n=22	-16.07 (86.81), n=23	p=0.98
Baseline to Placebo Δ: Nucleus Accumbens (beta values)	-16.24 (49.11), n=22	-19.08 (69.05), n=23	p=0.98
**Win Outcome**			
Baseline: Caudate (beta values)	91.81 (136.79), n=25	89.88 (192.72), n=25	p=0.97
Baseline: Putamen (beta values)	101.48 (144.76), n=25	171.76 (152.92), n=25	p=0.97
Baseline: Nucleus accumbens (beta values)	126.49 (175.89), n=25	190.69(228.13), n=25	p=0.97
Placebo: Caudate (beta values)	78.29 (153.17), n=24	69.51 (208.73), n=24	p=0.97
Placebo: Putamen (beta values)	44.93 (120.30), n=24	47.26 (166.41), n=24	p=0.97
Placebo: Nucleus accumbens (beta values)	33.28 (159.71), n=24	150.13 (143.33), n=24	p=0.099
Baseline to Placebo Δ: Caudate (beta values)	-24.29 (240.46), n=22	-33.79 (301.91), n=23	p=0.97
Baseline to Placebo Δ: Putamen (beta values)	-70.13 (190.49), n=22	-37.71 (196.88), n=23	p=0.97
Baseline to Placebo Δ: Nucleus Accumbens (beta values)	-99.02 (256.71), n=22	-66.17 (185.62), n=23	p=0.97

**Task Performance**			
Baseline: Overall accuracy (%)	41.89 (8.78), n=25	42.72 (7.61), n=25	p=0.87
Baseline: Reward trial accuracy (%)	49.83 (13.90), n=25	47.17 (12.01), n=25	p=0.87
Baseline: Overall reaction time (ms)	297.24 (36.52), n=25	318.61 (21.41), n=25	p=0.096
Baseline: Reward trial reaction time (ms)	289.35 (52.09), n=25	319.88 (23.34), n=25	p=0.096
Placebo: Overall accuracy (%)	40.56 (11.64), n=25	40.34 (8.89), n=24	p=0.94
Placebo: Reward trial accuracy (%)	49.00 (16.46), n=25	47.57 (1.84), n=24	p=0.87
Placebo: Overall reaction time (ms)	301.45 (28.35), n=25	313.36(24.24), n=24	p=0.45
Placebo: Reward trial reaction time (ms)	301.78 (30.72), n=25	309.05(25.07), n=24	p=0.87
Baseline to Placebo Δ: Overall accuracy (%)	0.00 (12.72), n=23	-1.69 (8.83), n=23	p=0.87
Baseline to Placebo Δ: Reward trial accuracy (%)	0.36 (16.99), n=23	1.27 (16.64), n=23	p=0.93
Baseline to Placebo Δ: Overall reaction time (ms)	0.59 (34.47), n=23	-5.46(27.82), n=23	p=0.87
Baseline to Placebo Δ: Reward trial reaction time (ms)	9.27 (56.25), n=23	-10.93 (34.43), n=23	p=0.45

p-values are from two-sided independent sample t-tests, with FDR correction for multiple comparisons. N/A: Unable to perform t-test as all values are 0.

Supplementary References

1. Alsop DC, Detre JA, Golay X, et al. Recommended implementation of arterial spin-labeled perfusion MRI for clinical applications: A consensus of the ISMRM perfusion study group and the European consortium for ASL in dementia. *Magn Reson Med*. Jan 2015;73(1):102-16. doi:10.1002/mrm.25197
2. Wang Z, Wang J, Connick TJ, Wetmore GS, Detre JA. Continuous ASL (CASL) perfusion MRI with an array coil and parallel imaging at 3T. *Magnetic Resonance in Medicine*. 2005;54(3):732-737. doi:https://doi.org/10.1002/mrm.20574
3. Knutson B, Fong GW, Adams CM, Varner JL, Hommer D. Dissociation of reward anticipation and outcome with event-related fMRI. *NeuroReport*. 2001;12(17):3683-3687.
4. Hagberg GE, Zito G, Patria F, Sanes JN. Improved detection of event-related functional MRI signals using probability functions. *Neuroimage*. Nov 2001;14(5):1193-205. doi:10.1006/nimg.2001.0880
5. Kim AJ, Grégoire L, Anderson BA. Value-Biased Competition in the Auditory System of the Brain. *Journal of Cognitive Neuroscience*. 2021;34(1):180-191. doi:10.1162/jocn_a_01785
