# Supplementary material for: Striatal dopamine D2/D3 receptor regulation of human reward processing and behaviour
Source: Nat Commun. 2025 Feb 21;16:1852. doi: 10.1038/s41467-025-56663-7 (PMC11845780; doi:10.1038/s41467-025-56663-7)
Supplement: Supplementary file 2 — Reporting Summary [file 41467_2025_56663_MOESM2_ESM.pdf]

nature portfolio

Corresponding author(s): Martin Osugo

Last updated by author(s): Jan 3, 2025

Reporting Summary

Nature Portfolio wishes to improve the reproducibility of the work that we publish. This form provides structure for consistency and transparency in reporting. For further information on Nature Portfolio policies, see our Editorial Policies and the Editorial Policy Checklist.

Statistics

For all statistical analyses, confirm that the following items are present in the figure legend, table legend, main text, or Methods section.

n/a	Confirmed	
☐	☒	The exact sample size (n) for each experimental group/condition, given as a discrete number and unit of measurement
☐	☒	A statement on whether measurements were taken from distinct samples or whether the same sample was measured repeatedly
☐	☒	The statistical test(s) used AND whether they are one- or two-sided<br>*Only common tests should be described solely by name; describe more complex techniques in the Methods section.*
☐	☒	A description of all covariates tested
☐	☒	A description of any assumptions or corrections, such as tests of normality and adjustment for multiple comparisons
☐	☒	A full description of the statistical parameters including central tendency (e.g. means) or other basic estimates (e.g. regression coefficient) AND variation (e.g. standard deviation) or associated estimates of uncertainty (e.g. confidence intervals)
☐	☒	For null hypothesis testing, the test statistic (e.g. F, t, r) with confidence intervals, effect sizes, degrees of freedom and P value noted<br>*Give P values as exact values whenever suitable.*
☒	☐	For Bayesian analysis, information on the choice of priors and Markov chain Monte Carlo settings
☒	☐	For hierarchical and complex designs, identification of the appropriate level for tests and full reporting of outcomes
☐	☒	Estimates of effect sizes (e.g. Cohen's d, Pearson's r), indicating how they were calculated

Our web collection on statistics for biologists contains articles on many of the points above.

Software and code

Policy information about availability of computer code

Data collection	MRI images were acquired on a 3T Siemens Magnetom Prisma. Software: VE11C; Siemens Siemens 3T Trio software: VB17
Data analysis	Symptom data were analysed in Matlab version 9.13.0.2049777 and SPSS v25, and plotted in GraphPad Prism (Version 10.2.3). Negative symptoms and extrapyramidal symptoms were analysed by fitting a linear mixed-effects model in Matlab using the function fitlme. The code was as follows: fitlme(Data, 'Response~DrugCondition + TreatmentOrder + Response_at_Baseline + (DrugCondition|subjectID)')<br><br>MRI data were analysed with FSL version 6.00.

For manuscripts utilizing custom algorithms or software that are central to the research but not yet described in published literature, software must be made available to editors and reviewers. We strongly encourage code deposition in a community repository (e.g. GitHub). See the Nature Portfolio guidelines for submitting code & software for further information.

Data

Policy information about availability of data

All manuscripts must include a data availability statement. This statement should provide the following information, where applicable:

- Accession codes, unique identifiers, or web links for publicly available datasets
- A description of any restrictions on data availability
- For clinical datasets or third party data, please ensure that the statement adheres to our policy

The conditions of the ethical approval of this study do not permit unrestricted access to the raw data. De-identified individual participant data are available for research purposes from the corresponding author (martin.osugo@kcl.ac.uk) from the publication date, subject to a data-sharing agreement, with the exception of data from a minority of subjects who did not consent to de-identified data being used to support future research. Requests will be responded to within 15 working days. The conditions of the ethical approval of the study stipulate that access to data which may allow identification of volunteers will only be permitted for research that has been independently reviewed by an ethics committee. Source data are provided with this paper.

Research involving human participants, their data, or biological material

Policy information about studies with human participants or human data. See also policy information about sex, gender (identity/presentation), and sexual orientation and race, ethnicity and racism.

Reporting on sex and gender	The sample demographics are reported in table 1. The study sample is broadly representative of the UK population, with 58% of the sample self-reporting as female, as compared to 51% of the UK population.<br>Sex and gender based analyses were not conducted as the study has a within-subject design, where each subject acts as their own control. The findings are therefore generalisable to both sexes. However, disaggregated numbers for individual experiments are provided in the source data files.
Reporting on race, ethnicity, or other socially relevant groupings	Ethnicity data was self reported according to categories set by the UK census. The sample is broadly representative of the general population in the UK. Ethnicity/race based analyses were not conducted as the study has a within-subject design, where each subject acts as their own control.
Population characteristics	50 subjects completed the study:<br>29 female, 21 male<br>mean age 26.6 (SD 8.15)<br>31 White, 9 Asian, 3 Black, 7 Mixed/Other ethnicity<br><br>All subjects were healthy volunteers aged 18-65 years, exclusion criteria were; history of psychiatric illness (including alcohol/ substance dependence) as determined by self-report and the Mini-International Neuropsychiatric Interview, current use of any illicit substances as determined by urine drug of abuse testing and self-report, first degree relative with a psychotic disorder, contraindications to dopamine antagonists/partial agonists or MRI scanning, current or significant previous use of psychotropic or dopamine modulating drugs, pregnancy or breastfeeding, participation in a study of unlicensed medicines within the previous 30 days, and presence of significant CNS disorder (e.g. significant head trauma, epilepsy etc.), medical disorder, or clinically relevant abnormal findings at the screening assessment, as determined by the principal investigator.
Recruitment	Healthy volunteers aged 18-65 years were recruited by public advertisement. Adverts were posted on both general websites and research volunteer circulars in order to recruit a diverse range of participants. As with all studies of this type, there may be a degree of selection bias but this is unlikely to alter the overall results on the role of D2/D3 modulation on reward function/behaviour.
Ethics oversight	This study was approved by the London – West London and GTAC NHS Research Ethics Committee (Ethics Committee Reference Number: 18/LO/1044).

Note that full information on the approval of the study protocol must also be provided in the manuscript.

Field-specific reporting

Please select the one below that is the best fit for your research. If you are not sure, read the appropriate sections before making your selection.

☒ Life sciences ☐ Behavioural & social sciences ☐ Ecological, evolutionary & environmental sciences

For a reference copy of the document with all sections, see nature.com/documents/nr-reporting-summary-flat.pdf

Life sciences study design

All studies must disclose on these points even when the disclosure is negative.

Sample size	This is an original study and there are no studies in the literature exploring the effect of one week of antipsychotics in healthy controls using MRI imaging as the primary outcome measure. However, in a similar MRI study in patients with schizophrenia, Nielsen et al demonstrated increase in the right ventral striatum activation over time following 6 weeks of antipsychotic treatment (effect size = 1) (Nielsen et al., 2012).

Based on this study, Using G*POWER, a sample size of 24 in each group would provide >95% power to detect this effect size using a paired t-test for alpha = 0.05 (two-tailed).

Data exclusions

Subjects with undetectable plasma drug levels following the active treatment week were excluded from all post baseline analyses. These subjects would not have contributed meaningful data to the conclusions of the study on the effects of D2/D3 modulators as there was no evidence of them taking D2/D3 modulators. These exclusions were pre-planned. Five subjects in the amisulpride sample and one subject in the aripiprazole sample were excluded for this reason.

Neuroimaging:

3 scans were excluded due to unusable MRI data. This consisted of 1 scan in the baseline condition and 2 in the amisulpride sample (both in the placebo condition). The data were unusable due to the structural MRI data being incorrectly exported, severe fMRI imaging artefacts and the participant having the response triggers in the wrong hands during functional imaging, respectively. The plan to exclude data for these reasons was pre-specified.

Scans were also excluded on the basis of task performance to ensure only data from participants who were actively and appropriately engaged in the task were included in the final analysis.
For the main comparison of drug vs placebo, 4 scans were excluded on this basis:
1 in amisulpride sample (in amisulpride condition)
3 in aripiprazole sample (1 in aripiprazole condition, 2 in placebo condition).
For the supplementary analysis of baseline task effects, five subjects were excluded on this basis (all scored 7% or less).

The plan to exclude subjects on this basis was pre-specified in the statistical analysis plan, but the exact threshold for exclusion was not defined in advance of the study commencing. The threshold of 2.5SDs away from the session average was set during the study (after collection of the amisulpride sample but before collection of the aripiprazole sample) and is consistent with prior analyses.

Replication

Not relevant

Randomization

Upon enrolment, subjects were randomised to treatment order (either amisulpride/aripiprazole or placebo first) by being assigned a unique study ID. Allocation of subjects was decided by the Latin Square (Williams design) controlling for order and first-order carry over effects.

Blinding

The study clinicians and subjects were blind to the treatment order during data collection (double-blind).

Reporting for specific materials, systems and methods

We require information from authors about some types of materials, experimental systems and methods used in many studies. Here, indicate whether each material, system or method listed is relevant to your study. If you are not sure if a list item applies to your research, read the appropriate section before selecting a response.

Materials & experimental systems

n/a	Involved in the study	
☒	☐	Antibodies
☒	☐	Eukaryotic cell lines
☒	☐	Palaeontology and archaeology
☒	☐	Animals and other organisms
☒	☐	Clinical data
☒	☐	Dual use research of concern
☒	☐	Plants

Methods

n/a	Involved in the study	
☒	☐	ChIP-seq
☒	☐	Flow cytometry
☐	☒	MRI-based neuroimaging

Plants

Seed stocks

N/A

Novel plant genotypes

N/A

Authentication

N/A

Magnetic resonance imaging

Experimental design

Design type	Event related fMRI design; MID task
Design specifications	The MID task probes brain activation upon expectation and receipt of monetary reward in a multiple-trial design. The task contains two trial types (24 win trials and 48 neutral trials). Participants are instructed to respond as quickly as possible to a target stimulus, and can win money if they respond quickly enough during win trials. The total amount of money to be won is £7.20, with £0 or £0.30 at stake in each trial. Each trial begins with the presentation of a cue stimulus for 500ms, which denotes whether the trial is a win trial (orange square) or a neutral trial (blue square). Following the cue, there is an anticipation period (interstimulus interval (ISI)) which varies randomly between 2, 3, and 4 seconds. The target stimulus (a white square) is then presented for a variable duration (starting at 300ms, 16.67ms subtracted or added each trial depending on performance in previous trial, range 200-400ms), during which time the subject has to respond. The target hit rate was approximately 50%. Following the target, feedback on the outcome of the trial is presented. The duration of feedback is also dynamic, to ensure that the total duration of the target plus the feedback is 1300ms. Following the feedback, an inter-trial interval (ITI) consisting of a fixation point is presented, which varies randomly between 2.2 and 10.2 seconds in one-second increments, on an approximately Poisson distribution.
Behavioral performance measures	Correct responses (binary outcome) during presentation of the target stimulus and reaction time of correct responses were collected. As described above, sessions where subjects scored more than 2.5SDs from the session mean for percentage of correct responses or reaction time in across all trials were excluded.

Acquisition

Imaging type(s)	Structural and functional
Field strength	3T
Sequence & imaging parameters	Structural and functional MRI images were acquired on a 3T Siemens Magnetom Prisma with a 64-channel head coil. High resolution T1 weighted volumes were acquired using a MP2RAGE sequence (176 slices, FOV read = 256 mm, TR = 5000.0 ms, TE = 2.98 ms, TI 1 = 707 ms, TI 2 = 2500 ms, flip angle 1 = 4°, flip angle 2 = 5°, 1 mm isotropic voxels, band-width = 240 Hz/pixel, echo spacing = 7.1 ms).<br>For the MID task, at each scanning session, 304 functional volumes were acquired using a BOLD echo planar imaging sequence (FOV read 220mm, 42 slices, slice thickness 3 mm, voxel size 3.4 x 3.4 x 3mm, TR 2400 ms, TE 30 ms). The first six volumes of each functional run were discarded to allow for T1 saturation effects.<br>Measurement of regional CBF was carried out using a 3D pseudo-continuous ASL (3D-PCASL) sequence and the following parameters: bolus duration= 1800 ms, inversion time= 3600ms, FOV= 220 mm, TR= 4000ms, TE= 13.32ms, slice thickness= 3 mm, resolution 1.7 x 1.7 x 3 mm, acquisition time= 4 minutes 42 s. Eight control-label pairs were used to derive a perfusion-weighted difference image. The sequence included background and fat suppression for optimal reduction of the static tissue signal. The sequence also included a perfusion calibration (M0) scan with TR= 5,000 ms to compute the CBF map in standard physiological units (ml blood/100 g tissue/min).1, 2
Area of acquisition	Whole brain

Diffusion MRI ☐ Used ☒ Not used

Preprocessing

Preprocessing software	Image processing was performed using FSL version 6.00. Anatomical images were pre-processed using the FSL_anat function in FSL to perform bias correction, transformation into a standard stereotactic space (MNI152) and brain extraction using the BET extraction tool.<br><br>Functional image series were pre-processed with a 100s high-pass filter, head motion correction, 6mm full width at half maximum spatial smoothing and co-registration to the T1-weighted structural image before transformation to standard space.<br><br>Quantified CBF maps for each subject and session were skull-stripped with BET as implemented in FSL, co-registered to their corresponding baseline structural scan, normalized to the study-specific T1-weighted template and subsequently warped to standard MNI space and resampled to a 2 mm3 isotropic resolution using ANTs. Finally, CBF images were spatially smoothed using a 6 mm FWHM Gaussian kernel.
Normalization	Registration to high resolution structural and standard space images was carried out using FLIRT in FSL 6.00. Registration from high resolution structural to standard space was then further refined using FNIRT nonlinear registration
Normalization template	MNI152
Noise and artifact removal	The times of the stimulus conditions were convolved with a gamma function to simulate the haemodynamic response function. Motion correction was performed using MCFLIRT.

Volume censoring | No volume censoring used

Statistical modeling & inference

Model type and settings

The FEAT module in FSL 6.00 was used to fit a generalised linear model.
First level analysis: Standard head-motion parameters were included in the first-level (subject-level) models as nuisance regressors. Temporal derivatives of task-related events, neutral hit and neutral miss explanatory variables were also included as nuisance regressors in the 1st level model
The second level analysis was a two-sided paired t-test comparing the amisulpride or aripiprazole condition and placebo conditions

Effect(s) tested

The contrasts of interest in the MID task were the subtraction contrasts of the reward anticipation period minus the neutral anticipation period (reward anticipation), and the reward outcome period minus the missed reward outcome period (reward outcome).

Specify type of analysis: ☐ Whole brain ☐ ROI-based ☒ Both

Anatomical location(s): Three bilateral striatal ROIs for the caudate, putamen, and nucleus accumbens were defined separately using the Harvard Oxford subcortical atlas, at a threshold of 50% probability.

Statistic type for inference
(See Eklund et al. 2016)

FSL's randomise tool with 5,000 permutations

Correction

FWE correction for multiple comparisons using threshold-free cluster enhancement at $p<0.05$.

Models & analysis

n/a	Involved in the study
☒	☐ Functional and/or effective connectivity
☒	☐ Graph analysis
☒	☐ Multivariate modeling or predictive analysis
